# Crotalism

**Published:** 1899-09

**Authors:** John J. Millar

**Affiliations:** Sioux City, Iowa


### COMMUNICATION.

### CROTALISM.

APPEARING in the JOURNAL OF COMPARATIVE MEDICINE AND VETERINARY ARCHIVES for August is an article entitled "Epizoötic Cellulitis Among Horses," by Dr. Manly, of Pittsburg, Kansas, and although the symptoms expressed are not very elaborate, still they sufficiently corroborate the symptoms of crotalism (or bottom disease) and due in every case to the eating of the rattle-weed (*Crotalaria saggitalis*), which is found in the meadows of South Dakota and Iowa, along the Missouri River bottoms.

The most noticeable, and perhaps the first symptom of crotalism is the refusal of grain, only eating very sparingly or not at all; later the animals affected will gape or yawn frequently, and this symptom is almost diagnostic, accompanied by a sudden spasmodic stamping of the hind foot, as if kicking at flies. Later a weaving gait, emaciation and death.

Throughout the course of the disease the temperature is normal or subnormal, and while in the stable the animal appears languid, oftentimes apparently sleeping while standing, with the head pressed lightly against the corner of the stall or manger, waking up suddenly to take a mouthful of old hay or straw, which is frequently retained in the mouth while another nap is enjoyed.

It is always the case, after the disease has passed on to an advanced stage, that the animal will lick out the dirt accumulated in the bottom of the manger, particularly the stems and pods of the *Crotalaria saggitalis*, which keeps adding fuel to the already emaciated system, until at last death relieves the victim.

JOHN J. MILLAR, D.V.S.,
Sioux City, Iowa.

